# Determinants of Neonatal Mortality in North Shoa Zone, Amhara Regional State, Ethiopia

**DOI:** 10.1371/journal.pone.0164472

**Published:** 2016-10-14

**Authors:** Tufa Kolola, Meseret Ekubay, Endalamaw Tesfa, Wogene Morka

**Affiliations:** 1 Institute of Medicine and Health Sciences, Debre Berhan University, Debre Berhan, Ethiopia; 2 College of Medicine and Health Sciences, Bahir Dar University, Bahir Dar, Ethiopia; Centre Hospitalier Universitaire Vaudois, FRANCE

## Abstract

In Ethiopia, neonatal mortality has been declined since the declaration of Millennium Developmental Goals, but the rate was slower since 2006. Thus, this study was designed to assess the determinants of neonatal mortality (NM) in North Shoa Zone. A community based case-control study was conducted on 84 cases and 252 controls. Cases were deceased new-borns within 28 days of birth while controls were infants survived beyond the first 28 days. Data were collected from mothers of the cases and controls using interviewer administered questionnaires. Multivariate analysis was done to examine determinants of NM. Variables significantly associated with NM in bivariate analysis were selected for multivariate analysis. Neonates whose mothers not attended antenatal care (AOR: 3.47; 95%CI: 1.44–8.32), delivered at home (AOR: 2.86; 95%CI: 1.56–5.26), and not received postnatal care services (AOR: 3.09; 95%CI: 1.73–5.51) were more likely to die. The odds of neonatal death was higher among neonates not breastfed within the first hour of delivery than those who breastfed within the first hour of delivery (AOR: 23.48; 95%CI: 8.43–65.37). Likewise, no-colostrum intake was positively associated with neonatal death. Neonates born to mothers who not received or received a single dose of tetanus toxoid injection (TTI) were more likely to experience death than those neonates born to mothers who received two or more doses of TTI (AOR: 2.05; 95%CI: 1.14–3.70). Furthermore, being small in size at birth (AOR: 2.66; 95%CI: 1.33–5.33) and male in sex (AOR: 1.85; 95% CI: 1.06–3.26) were risk factors for NM. In conclusion, neonatal mortality was significantly associated with factors that are modifiable through addressing the continuum-of-care approach in healthcare services in North Shoa. This implies that ensuring a continuity of health care services for maternal and new-borns from antenatal to postnatal care will improve neonatal survival.

## Introduction

Neonatal mortality refers to the infant deaths that occur during the first four weeks after birth [1]. Neonatal mortality has been declined in the whole regions of the world since the declaration of Millennium Developmental Goals (MDGs), but the progress has been the slowest in sub-Saharan Africa compared to other regions of the world [2]. In 2013, 2·761 million babies died worldwide in the neonatal period which accounted for 41·6% of under-5 deaths compared to 37.4% in 1990 [3, 4]. The greatest risk of this death was at the very beginning of life, 31·9% happened in the early neonatal period and 9·7% in the late neonatal period [4]. The highest numbers of deaths were registered in south-central Asian and sub-Saharan African countries [4, 5]. Ethiopia was ranked to the third position holding the highest number of neonatal deaths from Eastern sub-Saharan countries in 2013 [4], and regional differences were also observed in neonatal mortality with higher rate in Amhara Regional State(54/1000 live births) than most of other Regional States of Ethiopia [6].

Preterm birth, intra-partum related complications/birth asphyxia and severe infections have been identified as the three leading causes of neonatal deaths globally [3, 7, 8]. Universal coverage of health interventions, i.e. tetanus toxoid immunization, skilled attendance at birth, access to emergency obstetric care, immediate and exclusive breastfeeding, preventing hypothermia, and if needed, resuscitation, care of low birth weight infants, and treatment of infection, could avert up to 72% of all new-born deaths, but such interventions do not reach those most in need [7, 9].

Identifying the modifiable determinants of neonatal death is useful for designing intervention programmes that improve neonatal survival [10]. Several studies from developing countries have shown that neonatal mortality is influenced by physical accessibility to health facility [11, 12], antenatal care [13–15], domestic violence during pregnancy [13], pregnancy complications [15], tetanus toxoid immunization for the mothers [16–18], place of delivery [14, 17], delivery complications [18], breastfeeding within the first hour of delivery [13, 14], postnatal care [14], birth weight [11, 13, 14], gestational age [11, 13, 15], birth spacing [14, 18], sex of the child [11, 12, 18, 19], previous history of neonatal death [13], household wealth [12, 19], maternal age [18, 19], and educational status of the mothers [18]. Most of these studies were conducted at health facilities and hence subjected to selection bias to apply the results to the community. Therefore, this study is designed to assess the determinants of neonatal mortality using community-based case-control study. The results of this study provide useful insights in planning intervention programmes for neonatal survival in our study area and other similar settings of Ethiopia.

## Methods

### Study area and period

This study was conducted in North Shoa Zone of Amhara Regional State, Ethiopia from January to May 2015. The Zone has a total of 24 Districts, and Debre Berhan is the capital of this Zone; located 130 kilometres to the North of Addis Ababa. According to the information obtained from the Zonal Health Department, there are 6 hospitals, 92 health centers, and 387 health posts which are run by government. In addition, there are 2 hospitals and 65 clinics owned by the private owners in this Zone.

### Study design and sample

A community based unmatched case-control study was conducted on 84 cases and 252 controls. Cases were deceased new-borns within 28 days of birth while controls were infants survived beyond the first 28 days. Sample size was calculated using Open Epi statistical software considering 80% power of the study, 95% confidence level, proportion of the cases born to mothers with history of previous neonatal death was36%, proportion of the controls born to mothers with history of previous neonatal death was14%, the ratio of controls to cases is 3:1 and design effect of 2. Two stage sampling technique was used to select the study participants. In the first stage, eight Districts were selected out of 24 Districts using random sampling technique. In the second stage, study participants were recruited from those eight Districts that were randomly selected in the first stage. Accordingly, preliminary survey was carried out in those eight Districts to identify cases and controls. Following preliminary survey, all identified cases were recruited as sample of cases while controls were recruited using random sampling technique. The controls were selected from the respective communities that yielded the cases.

### Inclusion and Exclusion

Inclusion criteria for the cases were singleton new-born babies who died in the neonatal period (during the first 28 days of life) in 2014. Three controls were matched for each case on the basis of locality and year of birth. Surviving infants were included in this study as controls. Exclusion criteria were stillbirths, severely ill mothers and infants whose mothers died.

### Study variables

#### Dependent variable

The dependent/outcome variable is neonatal mortality.

#### Independent variables

Independent variables used in this study were: maternal educational level [no formal education, elementary school (grade1-8), high school and above (≥grade 9)], place of residence (urban, rural), maternal marital status (currently married, currently not married), household wealth index (low, medium, high), mother’s age at child birth in years (<20, 20–34, ≥35 years), child’s sex (male, female), child’s size at birth (small, normal), preceding birth interval (first birth, <2 years, ≥2years); birth order (first, 2nd- 3^rd^, 4th +), tetanus toxoid injection before birth (none or single dose of TTI, 2 or more doses of TTI), antenatal care utilization (Yes, No), frequency of antenatal care visit (1–3 visit, ≥4 visit), place of delivery (home, health facility), postnatal care utilization (Yes, No), breastfed within the first hour of delivery (Yes, No), fed colostrum without discard (Yes, No), previous neonatal death (Yes, No).

In this study, maternal marital status was dichotomized as currently married and currently not married. Currently married were those who were married and cohabiting with their husbands while currently not married were those who were never married, divorced or widowed. In settings where children are not often weighed at birth, the mothers’ report of the size of their babies at birth is used as the proxy for the child’s weight [6]. Accordingly, the data on the child’s size at birth were collected based on the five categories as very small, small, average, big, and very big. The child’s size at birth was then dichotomized as small and normal. We consider child’s size at birth is small if the mothers report for the child’s size at birth was either very small or small. On the other hand, we consider child’s size at birth is normal if the mothers report for the child’s size at birth was either average, big or very big.

The household wealth index was made based on the five categories of wealth quintile as lowest, second, middle, fourth, and highest. These categories of wealth quintile were further trichotomized as low, medium and high. The lowest and second wealth quintile merged into the low category, the middle and fourth merged into the medium category and the highest wealth quintile as high category.

### Data collection

Data were collected from mothers of the cases and controls using structured questionnaires which adopted from Ethiopian Demographic and Health Survey [6], and other similar study done elsewhere [14, 19]. The questionnaire (S1 Appendix) was pre-tested on similar settings which are not part of the study area and the necessary modifications were made on some items of the questionnaire. Moreover, data collectors (BSc holder in Nursing) were collected the data in local language (Amharic) through face-to-face interview using questionnaires.

### Data Analysis

Data entry and cleaning were carried out using Epi Info version 3.5.1 and analyzed by SPSS version 21. Bivariate and multivariate analyses were done using binary logistic regression model to examine determinants of neonatal mortality. Bivariate analysis was done first and then followed by multivariate analysis, as in previous studies [19, 20]. The bivariate analysis was done to examine the odds of exposure difference between cases and controls, and unadjusted odds ratios with 95% confidence interval were calculated for all variables included in the bivariate model. The multivariate analysis was done to examine the individual effects that each explanatory variable has on neonatal mortality after adjusting for other variables that entered into the multivariate model. Variables significantly associated with neonatal mortality at p-value <0.05 in the bivariate analysis were entered into the multivariate model and adjusted odds ratios with 95% confidence interval were calculated.

### Ethical consideration

The proposal of this study was ethically reviewed and approved by the Ethics Committee of Institute of Medicine and Health Sciences of Debre Berhan University. Then ethical clearance letter was obtained from this Institute and submitted to Zonal Health Department for permission to undertake the study. In addition, verbal informed consent was obtained from all study participants. Verbal consent was obtained because majority of our study participants cannot read and write to provide them with written informed consent. Moreover, no personal identifiers were used on data collection questionnaire and data that obtained from the study participants were kept confidentially.

## Results

### Determinants of neonatal mortality

In this study, a total of 84 cases and 252 controls were enrolled to assess determinants of neonatal mortality. The results of bivariate analysis reveals that household wealth, antenatal care visit, frequency of antenatal care visit, place of delivery, postnatal care visit, breast feeding within the first hour of delivery, feeding colostrum, tetanus toxoid injection before birth of last baby, child’s size at birth and child’s sex were significantly associated with neonatal mortality. On the other hand, mother's age at birth, place of residence, maternal marital status, educational status of the mothers, history of previous neonatal death, preceding birth interval and birth order were not associated with neonatal mortality (Tables 1 and 2).

**Table 1:** Bivariate logistic regression analysis depicting the association between independent variables and neonatal mortality in North Shoa Zone of Amhara Regional State, 2014.

**Variables**	**Cases (N = 84)**	**Controls (N = 252)**	**COR (95%CI)**	**p-value**
Mother's age at birth				
<20	5	8	2.22(0.58–8.49)	0.243
20–34	70	212	1.17(0.53–2.58)	0.690
≥35	9	32	1	
Place of residence				
Urban	21	79	1	
Rural	63	173	1.37(0.78–2.40)	0.271
Maternal marital status				
Currently married	73	228	1	
Currently not married	11	24	1.43(0.67–3.06)	0.355
Maternal education				
No formal education	32	98	1.46(0.73–2.90)	0.282
Grade 1–8	37	87	1.90(0.96–3.75)	0.064
High school and above	15	67	1	
Household wealth index				
Low	53	119	2.23(1.15–4.31)*	**0.017**
Medium	17	63	1.35(0.62–2.96)	0.455
High	14	70	1	
Antenatal care visit				
Yes	72	241	1	
No	12	11	3.65(1.55–8.62)*	**0.003**
Frequency of ANC visits	(N = 72)	(N = 241)		
1–3	46	119	1.81(1.05–3.12)*	**0.032**
4 or more	26	122	1	
Place of delivery				
Home	37	50	3.18(1.87–5.41)*	**0.000**
Health facility	47	202	1	
Post natal care visit				
Yes	33	179	1	
No	51	73	**3.79(2.26–6.35)** *	**0.000**
Breastfed within the first hour of delivery				
Yes	38	246	1	
No	46	6	49.62(19.84–24.14)*	0.000
Fed colostrum				
Yes	44	240	1	
No	40	12	18.18(8.84–37.38)*	**0.000**

*Statistically significant at p<0.0 after adjusted for other variables, and ANC- Antenatal care

**Table 2:** Bivariate logistic regression analysis depicting the association between independent variables and neonatal mortality in North Shoa Zone of Amhara Regional State, 2014.

**Variables**	**Cases (N = 84)**	**Controls (N = 252)**	**COR (95%CI)**	**p-value**
Previous neonatal death				
Yes	12	25	1.51(0.72–3.16)	0.271
No	72	227	1	
Preceding birth interval				
First birth	26	84	0.99(0.54–1.58)	0.770
<2years	4	7	1.70(0.48–6.05)	0.410
≥2years	54	161	1	
Tetanus toxoid injection				
None/a single dose of TTI	24	40	2.12(1.19–3.79)*	**0.011**
2 or more TTI	60	212	1	
Child’s size at birth				
Small	19	22	3.06(1.56–5.99)*	**0.001**
Normal	65	230	1	
Child’s sex				
Male	57	132	1.92(1.14–3.23)*	**0.014**
Female	27	120	1	
Birth Order				
First	23	82	0.65(0.34–1.24)	0.189
2^nd^- 3^rd^	33	105	0.73(0.40–1.32)	0.296
4^th^ +	28	93	1	

*statistically significant at p<0.05 before adjusted for other variables,

COR- Crude odds ratio, CI- Confidence interval, and TTI- Tetanus toxoid injection

In the multivariate analysis, the odds of dying during neonatal period was higher among neonates born to mothers who did not attend antenatal care (ANC) compared to neonates born to mothers who attended antenatal care (AOR: 3.47; 95%CI: 1.44–8.32). Neonates born at home were 2.86 as likely to die within the first 28 days of life compared with those born at health facility (AOR: 2.86; 95%CI: 1.56–5.26). The odds of neonatal death was three fold higher among neonates whose mothers not received postnatal care (PNC) services than the mothers who received postnatal care services (AOR: 3.09; 95%CI: 1.73–5.51).

The odds of neonatal death was much higher among those neonates who were not breastfed within the first hour of delivery than in those who were breastfed within the first hour of delivery (AOR: 23.48; 95%CI: 8.43–65.37). Likewise, neonates who were not fed colostrum were more likely to experience death during their first 28 days of life than that of who were fed colostrum (AOR: 3.91; 95%CI: 1.52–10.03). Mothers who not received or received a single dose of tetanus toxoid injection were more likely to lose their babies during neonatal period than that of mothers who received two or more doses of tetanus toxoid injection (AOR: 2.05; 95%CI: 1.14–3.70). Moreover, being small in size at birth (AOR: 2.66; 95%CI: 1.33–5.33) and being male in sex (AOR: 1.85; 95%CI: 1.06–3.26) were also identified as a risk factors for neonatal mortality. However, the multivariate analysis result reveals that neonatal mortality was independent of household wealth status and frequency of ANC visit (Tables 3 and 4).

**Table 3:** Multivariate logistic regression analysis depicting the independent predictors of neonatal mortality in North Shoa Zone of Amhara Regional State, 2014.

**Variables**	**Cases (N = 84)**	**Controls (N = 252)**	**AOR(95%CI)**	**p-value**
House hold wealth index				
Low	53	119	1.47(0.70–3.09)	0.315
Medium	17	63	1.29(0.56–2.97)	0.544
High	14	70	1	
Antenatal care visit				
Yes	72	241	1	
No	12	11	3.47(1.44–8.32)*	**0.005**
Frequency of ANC visits				
1–3	46	119	1.71(0.98–3.00)	0.060
4 or more	26	122	1	
Place of delivery				
Home	37	50	2.86(1.56–5.26)*	**0.001**
Health facility	47	202	1	
Post natal care visit				
Yes	33	179	1	
No	51	73	3.09(1.73–5.51)*	**0.000**

*Statistically significant at p<0.0 after adjusted for other variables,

ANC- Antenatal care

**Table 4:** Multivariate logistic regression analysis depicting the independent predictors of neonatal mortality in North Shoa Zone of Amhara Regional State, 2014.

**Variables**	**Cases (N = 84)**	**Controls (N = 252)**	**AOR(95%CI)**	**p-value**
Breastfed within the firsthour of delivery				
Yes	38	246	1	
No	46	6	23.48(8.43–65.37)*	**0.000**
Fed colostrum				
Yes	44	240	1	
No	40	12	3.91(1.527–10.03)*	**0.005**
Tetanus toxoid injection				
None or a single dose of TTI	24	40	2.05(1.14–3.70)*	**0.017**
2 or more TTI	60	212	1	
Child’s size at birth				
Small	19	22	2.66(1.33–5.33)*	**0.006**
Normal	65	230	1	
Child’s sex				
Male	57	132	1.85(1.06–3.26)*	**0.031**
Female	27	120	1	

*Statistically significant at p<0.0 after adjusted for other variables,

AOR- Adjusted odds ratio, CI- Confidence interval, and TTI- Tetanus toxoid injection

## Discussion

This study shows that neonates born to mothers who were not utilized antenatal, delivery and postnatal care services were more likely to experience death than those born to mothers that utilized those services. Consistent findings were reported from previous studies [11, 13, 14, 21, 22]. It is recommended that pregnant women without pregnancy complication should have at least four antenatal care visits to reduce the risk of neonatal mortality [23]. The risk reduction for neonatal mortality was more apparent when antenatal care visit is accompanied with safe delivery and postnatal care [24].

In this study, mothers who not received or received a single dose of tetanus toxoid injection were more likely to lose their babies during neonatal period than those mothers who received two or more doses of tetanus toxoid injection. In low and middle income countries, the excess risk of neonatal death was attributable to lack of at least two doses of tetanus toxoid vaccination during pregnancy [16, 17, 25]. Women who not received previous tetanus toxoid injection (TTI) need two doses of TTI during pregnancy; indeed, two or more doses of tetanus toxoid injection are recommended to assure high protective antibody against neonatal tetanus [17].

The findings of our study shows that the risk of neonatal death was in excess among neonates who were not breastfed within the first hour after delivery compared with those who were breastfed. Several studies conducted elsewhere also confirmed the protective effect of breastfeeding especially when initiated immediately after birth [13, 26–28] as recommended by UNICEF and WHO [6, 29]. This study also shows that fail to feed colostrum after birth was significantly associated with an increased odds of neonatal mortality. Colostrum, i.e. the first liquid come out from the breast, is very important for the new-born babies to protect them from infections as it is rich in antibodies and white cells [30].

Neonates with small in size at birth were at an increased risk of death compared with neonates with normal birth weight at birth. Other studies have been consistently reported similar findings [8, 10, 11, 13, 14, 31, 32]. This can be explained as; low birth weight babies are more likely to born prematurely and suffer from infections [8, 31, 33, 34]. As a result, low birth weight could be considered as an enabling factor for neonatal mortality [31, 35].

Male neonates had higher odds of death compared to female neonates, which agree with previous findings from both developed and developing countries [8, 11, 18–20, 36, 37]. This could be due to the fact that neonatal period is the time at which innate biological differences between males and females are prominent. Males are more susceptible to both infections and conditions associated with prematurity and development than females [37–39]. Thus, biological differences are the prominent factor that imbalance mortality between male and female infants during their early life [7, 37, 40].

### Strengths and limitations of the study

The strength of this study is that, cases and controls were selected from same source population. As a result, cases and controls were comparable and representative of the source population to draw an inference. In addition, one case was compared with three controls on the basis of locality and year of birth to increase the power of study.

This study is not free from limitations. Information on neonatal mortality obtained only from surviving mothers, hence, liable to selective survival bias. Since children are not often weighed at birth in our study setting, mothers’ report of the size of baby at birth was used in five categories, i.e. very small, small, average, big, and very big, as proxy for the child’s weight. The child’s size at birth was then classified as small and normal birth weight. As a result, misclassification of birth weight might be possible. As significant numbers of neonates died so early without breastfeeding, the observed association between breastfeeding and neonatal mortality might have resulted in an overestimation of the true association. Furthermore, in a community-based retrospective study like case-control study, it is impossible to have data on some important variables. In the current study, we also fail to collect complete data on some important variables such as pregnancy complications, delivery complications, hypothermia and maternal nutrition, and consequently could not evaluate as determinants of neonatal mortality.

## Conclusions

This study identified several determinants of neonatal mortality which can serve to design strategies for decreasing neonatal mortality. Health care related factors such as antenatal care, tetanus toxoid injection during pregnancy, place of delivery and postnatal care are remained as determinants of neonatal mortality. Breastfeeding within the first hour of delivery, colostrum intake by the new-borns, sex of the child and size of the child at birth are also among the predictors of neonatal mortality in North Shoa. This suggests that strategies that ensure the uptake of the continuum of health care services for maternal and new-borns spanning from antenatal, to delivery, immediate neonatal and postnatal care are needed to reduce neonatal mortality in North Shoa.

## Supporting Information

S1 Appendix Blank copy of the questionnaire in English. (DOCX)

S1 Data Data set underlying the findings of this paper in SPSS format. (SAV)
