# Supplementary material for: Determinants of Neonatal Mortality in North Shoa Zone, Amhara Regional State, Ethiopia
Source: PLoS One. 2016 Oct 14;11(10):e0164472. doi: 10.1371/journal.pone.0164472 (PMC5065191; doi:10.1371/journal.pone.0164472)
Supplement: S1 Appendix — (DOCX) [file pone.0164472.s001.docx]

**Appendix I. Information sheet and Informed consent statements**

**Information sheet**

Introduction: Hello! Sir/Madam my name is ___________and I came from_________________. We are conducting a study on ‘Determinants of neonatal mortality in North Shoa Zone`. The aim of this study is to determine factors that affect neonatal mortality. The results of this study will be helpful to plan appropriate interventions that improve the survival of infants during their neonatal period.

**Consent statements (to be read by data collector for the study participants)**

I am going to ask you questions about factors that considered affecting neonatal death. You are randomly selected to participate in the study. Your participation in this study doesn’t involve any direct risk or benefit for you, but is very useful to improve the survival of neonates in this area. Your name will not be appearing on this questionnaire, and all the information you provide me will be strictly confidential. It takes 30 minutes to finish the interview and you are not obliged to answer any question you don’t wish to answer, and you can also end this interview at any time, if you wish to do so. Would you like to participate in the study?

Yes________ No_________

Interviewer, if the answer is “Yes” please let participant to sign (i.e. either using their signature or fingerprint) below to certify her verbal consent to take part voluntarily in the study. Otherwise, thank the client, conclude the conversation and file the questionnaire.

Signature_________

Date _____________ Identification No.__________

Contact address of principal investigator: - mobile: 0921777954

email:tufabest@gmail.com

Appendix II: **Questionnaire in English version**

**Part I: Socio- economic variables**

| S. No | Questions | Answers | Skip to |
| --- | --- | --- | --- |
| 101 | Age of the mother at delivery? | …………………in years |  |
| 102 | Place of residence | Urban…………………1  Rural………………….2 |  |
| 103 | Religion of the mother | Orthodox Christian …..1  Muslim………………..2  Protestant……………..3  Others specify…...…… |  |
| 104 | Ethnicity of the mother | Amhara……………..…1  Oromo…………………2  Tigrie…………………..3  Others specify………… |  |
| 105 | Marital status of the mother | Single…………………..1  Married…………………2  Divorced …………….…3  Widowed ………….……4 |  |
| 106 | Maximum educational level of the mother attended? | ------------------------------ |  |
| 107 | Maximum educational level of the father attended? | ------------------------------ |  |
| 108 | Maternal occupation status? | Farmer …………………1  Government employed…2  Private/NGO employed...3  Merchant ………………4  House wife ……………..5  Student…………….……6  Daily laborer……………7  Others specify ………… |  |
| 109 | Paternal occupation status? | Farmer …………………1  Government employed…2  Private employed………3  Merchant ……………....4  Daily laborer …..………5  Student…………….……6  Others specify ………. … |  |
| 110 | Which property do you have? (Circle all that you have) | Radio….....…..................1  Television………………2  Cow and ox ...………..…3  Sheep’s and goats…...…..4  Car/ Bajaj…….…….……5  Other specify…..……..… |  |
| 111 | Is the home you are living in your own? | Yes………………………1  No………………………..2 |  |
| 112 | Did you have a separate room for animals (if there) | Yes……………………….1  No………………………..2 |  |
| 113 | What is your monthly income in Ethiopian Birr or in item? | …………………………….. |  |
| 114 | Did you wash your hand with soap before breast feeding your baby? | Yes………………………1  No………………………..2 |  |
| 115 | Is there health institution in your vicinity? | Yes………………………1  No………………………..2 | If 2 go to 118 |
| 116 | What type of health institution is available in your vicinity? (it is possible to choose more than one) | Health post……………….1  Health center……………..2  Hospital…………………..3  Private clinic……………..4 |  |
| 117 | What is the average distance from your village to the health institution? (health center/ hospital) | ______________km |  |
| 118 | Is there a road that allows for the entrance of ambulance to your kebele? | Yes……………..…………1  No………………..………..2 |  |

**Part II: Neonatal Information**

| NO | Questions | Answers | Skip to |
| --- | --- | --- | --- |
| 201 | Age of your recent baby | ……………..in days |  |
| 202 | Sex of your recent baby | Male ………………………1  Female ……………………..2 |  |
| 203 | Did your recent baby vaccinated immediately right birth (Polio & BCG) | Yes…………………………1  No…………………………..2 |  |
| 204 | Birth space between your recent birth and the older (if there) | ______________ in years |  |
| 205 | Weight of your neonate at birth? | …………………in Kg  I don’t remember………...99 |  |
| 206 | Did you breast feed your recent baby within the first hour of delivery? | Yes.………………………1  No……….………………..2 |  |
| 207 | Did you feed your recent baby immediately without discarding the colostrum? | Yes……..……..………….1  No ………..………………2 |  |
| 208 | Did you feed your baby any liquids using a bottle? | Yes …………………..……1  No …………………………2 |  |
| 209 | Did do give additional foods for your neonate within 28 days of birth? | Yes …………………….…1  No ………………………...2 |  |
| 210 | Did you apply anything on the cord after delivery? | Yes ……………………….…1  No ………………………..….2 |  |
| 211 | Was the baby placed in SKIN-to-SKIN contact in the first 24 hours after delivery? | Yes ……………………….….1  No ………………………..…..2 |  |
| 212 | Time of first bath for your recent baby after delivery? | before 24 hours ………………1  After 24 hours ……………….. 2 |  |

**Part III: Maternal Information and Service Utilization**

| S. No | Questions | Answers | Skip to |
| --- | --- | --- | --- |
| 301 | Your family size in number? | ………………… |  |
| 302 | How many times you became pregnant? | -------------- |  |
| 303 | How many alive children did you have? | --------------- |  |
| 304 | Did you want your recent pregnancy? | Yes…………….………1  No ……………..………2 |  |
| 305 | When was your LMP and date of delivery for your recent delivery in Ethiopian calendar? | LMP…../……/…… Date of Birth …/ …/….  If you don’t remember at in what month did you give birth……… |  |
| 306 | Have you had ANC follow up for your most recent delivery? | Yes…………….………1  No ……………..………2 | If2 skip to 313 |
| 307 | If Q306 yes, how many times you attended ANC care for your most recent delivery? | ……………………………. |  |
| 308 | Where did you receive your ANC follow up for your most recent ANC visit? | Hospital .............................1  Health center.......................2  Private Hospital /Clinic.......3  Health Post ………..............4 |  |
| 309 | What was your gestational age at first ANC visit for your most recent pregnancy? | ……………………in month |  |
| 310 | Did you take a tablet during ANC follow up for you recent pregnancy to prevent anemia? | Yes………………………..1  No……..…………………..2 |  |
| 311 | Did you receive a vaccine to prevent tetanus for your recent pregnancy? | Yes…………..……………..1  No…………………………..2 |  |
| 312 | If Q311 yes, how many times did you receive TT immunization? | ………………………………. |  |
| 313 | Size of your recent baby at birth? | Very small…………………1  Small……………………….2  Average…………………….3  Large……………………….4  Very large…………………..5 |  |
| 314 | Your nutritional status during your recent pregnancy? | Less than usual ......................1  Same as usual ........................2  More than as usual………….3 |  |
| 315 | Did you prepare place of delivery for your recent delivery? | Yes……..……….…………..1  No ……………….………….2 |  |
| 316 | Where you gave birth for your most recent delivery? | Home ……………….….…...1  Heath center………………....2  Hospital ……………………..3 | If 2 & 3 go to 317 |
| 317 | Mode of delivery for your recent delivery? | Spontaneous vaginal delivery...1  Instrumental delivery…………2  Cesarean section………………3 |  |
| 318 | Duration of labour for your recent delivery? | …………………in hour |  |
| 319 | Did you receive postnatal care for your recent delivery? | Yes…………….………….1  No.………..……………….2 |  |
| 320 | If Q319 yes, how many times? | …………………………….. | End |

**Thank you for your time**
